# Unveiling population structure in South Pacific albacore: insights from genetics and growth

**DOI:** 10.1038/s41598-025-20480-1

**Published:** 2025-10-17

**Authors:** Dongqi Lu, Qinqin Lin, Feng Wu, Jiangfeng Zhu, Fan Zhang

**Affiliations:** 1 College of Marine Living Resource Sciences and Management, Shanghai Ocean University, Shanghai, 201306 China; 2 Key Laboratory of Sustainable Exploitation of Oceanic Fisheries Resources, Ministry of Education, Shanghai, 201306 China

**Keywords:** Population structure, Genome-wide resequencing, Albacore, Adaptive analysis, Growth, Marine biology, Conservation biology

## Abstract

Albacore (*Thunnus alalunga*) is a high-value, highly migratory tuna in the South Pacific that is currently assessed and managed as a single stock. Uncertainty it remains over whether southwestern and southeastern individuals constitute the same biological population, with implications for stock assessment and management. Here, we tested for population structure by combining genome-wide resequencing (from which a panel of putatively adaptive SNPs was derived) with analyses of growth (length-weight relationships and growth parameter estimates). Genetic analyses using adaptive markers revealed differentiation between western (WCPO) and eastern Pacific samples. Growth analyses indicated regional differences: western individuals attain larger asymptotic sizes (L∞), whereas eastern individuals exhibit faster growth (higher k) and different length-weight scaling (b). Together, the results provide moderate genomic evidence and strong phenotypic evidence for spatially structured variation in South Pacific albacore, consistent with a mixture of partially differentiated subpopulations. Further broad-scale sampling and integrative studies (e.g., neutral and adaptive genomics, otolith chemistry, tagging, and age-structured growth models) are needed to disentangle environmental and genetic drivers and to evaluate alternative stock-structure hypotheses for management.

**Supplementary Information:**

The online version contains supplementary material available at 10.1038/s41598-025-20480-1.

## Introduction

Metapopulations are a series of population units with a degree of connectivity^1^, which are commonly observed in marine fish species^2^. These local populations may be affected by different environmental and anthropogenic factors, leading to varying life history traits (i.e., recruitment, body growth, maturation)^3^. In marine fisheries, management units are often used to define different populations, which may conflict with the underlying genetic population structure of the species. Moreover, compared to the static management boundaries, population distribution often varies over time due to climate and ecosystem changes^4^, resulting in a mismatch between management and population boundaries.

Mis-specifying population structure increases risks of fishery management and can lead to overestimation of true harvest potential^5^. For example, a comparison of two models for Atlantic cod showed that assuming a single population per management unit—rather than the three genetically defined populations—can inflate estimates of productivity and resilience^6^, underscoring the importance of correctly defining population structure in management^7^. Ying et al.^2^ tested three population-structure scenarios and found that ignoring connectivity or treating regions as a single unit can cause overexploitation or local depletion. Conventional stock assessments struggle to detect and incorporate subpopulation status without a clear understanding of underlying structure, which increases the risk of population collapse. Fishing effort often does not adjust to the presence of subpopulations, and once biomass declines from overfishing it is difficult to rebuild and re-stabilize. For instance, overlooking small spawning components of Atlantic herring contributed to fisheries collapse^6,8^, and failure to account for shifts and heterogeneity in population dynamics played a central role in the northern cod collapse^5^. Accurate assessment of local dynamics therefore requires substantial resources to both identify structure and monitor its temporal changes— a task made especially difficult for highly migratory species.

Albacore is an ecologically and economically important tuna species that is widely distributed in the temperate regions of all three oceans^9^, accounting for about 5% of the global tuna catch^10^. Albacore are managed as six separate stocks: one each in the Mediterranean Sea and Indian Ocean, and two each in the Atlantic and Pacific Oceans, with the latter separated by the equator^9^. Albacore can grow to over 120 cm and 30 kg, with a longevity of up to 15 years. Due to its slow growth pattern^11^, albacore requires 4 to 5 years to reach maturity^12^, making it more susceptible to overfishing compared to tropical tuna species^13^. According to currently accepted knowledge, albacore in the South Pacific are believed to spawn in the Western and Central Pacific Ocean(WCPO) between 10°S and 25°S during summer^9^. Juveniles are primarily found in high-latitude regions, and their northward dispersal has been demonstrated by tagging data^9^. This stock supports important tuna fisheries, which accounted for 33.66% of the global albacore catch^10^. The South Pacific is managed by the Western and Central Pacific Fisheries Commission (WCPFC) and the Inter-American Tropical Tuna Commission (IATTC)^12^. The current assessment and management assume the South Pacific albacore as a single population, without considering potential spatial differences in biological parameters^14^.

Improved understanding of population structure is essential to further support management of albacore in the South Pacific. Currently, several lines of evidence suggest the potential presence of population structure^9,13,15,16^. Williams et al.^9^ and Farley et al.^17^ found distinct growth patterns between individuals from the central and western Pacific based on morphological evidence of gonad weights and length-at-age. Similarly, Macdonald et al.^16^ found that individuals from French Polynesia may originate from other spawning grounds, as suggested by otolith chemistry analysis. Previous genetic studies on South Pacific albacore have produced mixed results regarding population structure. Montes et al.^15^ found no significant differentiation at neutral loci between southeast and southwest Pacific samples, consistent with Laconcha et al.^18^, who used coding-region SNPs. By contrast, Anderson et al.^13^ —one of the few multi sample studies—detected divergence at adaptive SNPs, though geographic and temporal sampling uncertainty complicated interpretation. More recently, Vaux et al.^19^ identified north–south Pacific differentiation via a similar approach. Despite these advances, the link between observed genetic differentiation and potentially distinct spawning grounds remains unexplored. This gap underscores the need for integrated studies combining genetic, biological, and ecological data to elucidate connectivity and inform management.

While most prior work has focused on the WCPO and therefore provides regionally constrained evidence for population structure, our study extends sampling across the entire South Pacific. By explicitly including specimens from the eastern Pacific, we fill a critical geographic gap and provide a more comprehensive assessment of population differentiation. By integrating genome-wide re-sequencing data with regional growth comparisons— such as length-weight relationships and growth functions— we test for potential population structure of in South Pacific albacore using both genetic and growth-based analysis.

## Materials and methods

### Sampling

**Fig. 1:**
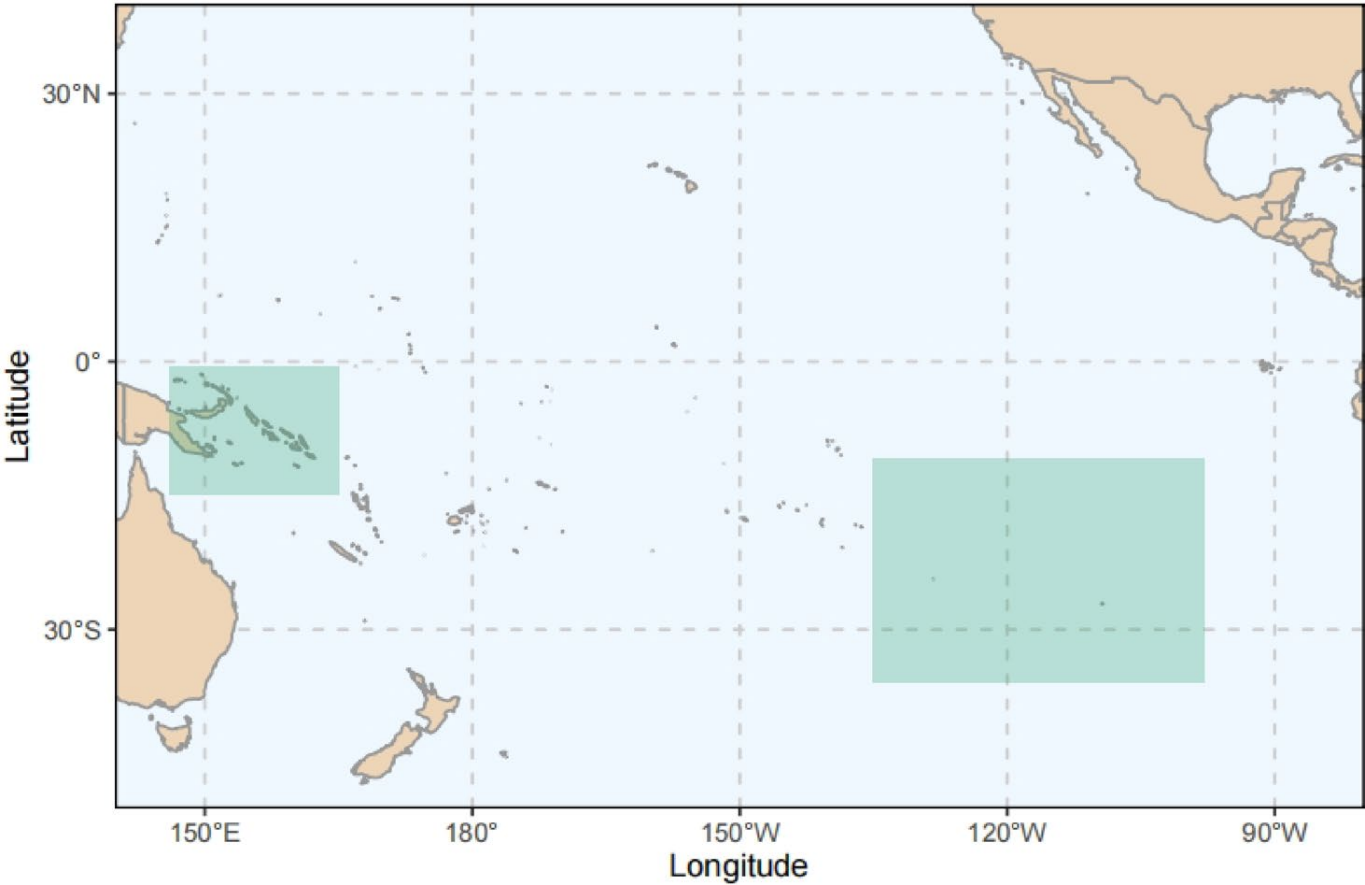
Sampling regions. Fishing areas for eastern samples (2021–2024) and western samples (2021–2023).

A total of 1053 frozen albacore (*N* = 1053) were sampled in the western (*N* = 288) and eastern (*N* = 765) Pacific Ocean, with corresponding sample sizes (Fig. 1). Western Pacific samples (146°E-165°E, 0.5°S-14.75°S) were collected in 2021, 2023 and 2024, while eastern samples (135°W-98°W, 11°S-36°S) were collected annually from 2021 to 2024. All samples were obtained from the commercial longline fisheries.

Size data were measured for all samples at the port. For the western samples, fork length (FL) was measured to the nearest 0.1 cm for all samples (*N* = 288), with a size distribution ranging from 77.1 to 103.6 cm FL. For the eastern albacore (*N* = 765), 66 specimens were measured only for FL, 239 specimens were measured only for anal length (AL) (to the nearest 0.1 cm) due to bleeding treatment, and 460 samples were measured for both FL and AL. For the 239 specimens without FL, a linear FL-AL conversion model (FL = 1.48*AL + 8.18; *N* = 460, R^2^ = 0.78) based on 460 specimens with paired FL and AL was applied to estimate FL. After conversion, the size distribution of eastern samples ranged from 64.6 to 105.1 cm FL.

The total weight (W) of each albacore was measured to the nearest 0.01 kg. FL-W parameters were estimated using 258 western and 453 eastern individuals with both measured FL and W, after excluding 30 western and 73 eastern samples due to ice coverage or body damage, and 239 eastern samples with converted FL.

For age determination and genetic analysis, caudal vertebrae were collected and frozen for further analysis. Age was successfully estimated for 228 western and 419 eastern specimens, while 60 western and 346 eastern samples were excluded due to insufficient processing or unclear growth increments. Among the aged eastern samples, 194 have measured FL, while the FL of the remaining 225 aged samples was converted from the formula above. To ensure consistency in the harvesting period (May to August 2021), muscle samples from caudal vertebrae of 23 western and 31 eastern samples were randomly collected and preserved at -80℃ for genetic analysis. Detailed information on samples used in each analysis is provided in Supplementary Table S1.

All sampled fish were dead upon collection at the port. Vessel trips were produced using the “maps” and “metR” packages for R (version 4.3.1) with the projection coordinate system GCS_WGS_1984. Spatial distribution of longline albacore catches from 2012 to 2023 was plotted using public-domain 5° × 5° gridded catch data from the WCPFC (https://www.wcpfc.int/public-domain) and IATTC (https://www.iattc.org/en-US/Data/Public-domain).

### Molecular analysis

#### Genomic DNA library preparation and sequencing

DNA was extracted from the muscle using the CTAB method. The quality of isolated genomic DNA was verified by using these two methods in combination: (1) DNA degradation and contamination were monitored on 1% agarose gels. (2) DNA concentration was measured by ND-2000 (NanoDrop Technologies). Only high-quality DNA sample (OD260/280 = 1.8 ~ 2.0, OD260/230 ≥ 2.0) was used to construct the sequencing library.

A total amount of 0.5 µg DNA per sample was used as input material for the DNA library preparations. The sequencing library was generated using Truseq Nano DNA HT Sample Prep Kit (Illumina USA) following the manufacturer’s recommendations and index codes were added to each sample. Briefly, the genomic DNA sample was fragmented by sonication to a size of 350 bp. Then, DNA fragments were end-polished, A-tailed, and ligated with the full-length adapter for Illumina sequencing, followed by further PCR amplification. After PCR products were purified (AMPure XP system), libraries were analyzed for size distribution by Agilent 2100 Bioanalyzer and quantified by real-time PCR (3nM). Paired-end DNA-seq sequencing library was sequenced with the Illumina NovaSeq system at Shanghai Majorbio Bio-pharm Technology Co., Ltd.

#### Variant discovery

Raw reads of low quality (mean phred score < 20), including reads containing adapter contamination and unrecognizable nucleotide (N base > 10), were trimmed or discarded by the software Fastp^20^. Reads after trimming were mapped to the reference genome of south bluefin tuna (*Thunnus maccoyii*) using BWAMEME software^21^ under default mapping parameters.

As the modified GATK Best Practise^22^, the alignment bam files were sorted by samtools^23^ and PCR duplicates were marked by MarkDuplicated. After doing the base quality recalibration, the germline variants calling, which contained SNPs across all samples using the Haplotyper and Gvcftyper programs in Sentieon genomics tools^24^. Variants were filtered using standard hard filtering parameters according to the GATK Best Practices pipeline.

All of the variants were annotated using SnpEff^25^. SNPs were categorized based on their positions on the chromosome (including intergenic regions, exons, introns, splicing sites, untranslated regions, and 1-kb upstream and downstream regions) and on their effects (including missense, start codon gain or loss, stop codon gain or loss and splicing mutations).

Before further analysis, several filtering steps were then performed to reduce false positives for SNPs and genotype calling: (i) remove SNPs with more than two alleles, (ii) remove SNPs with mean depth values over all samples less than 4, (iii) remove SNPs with minor allele frequency < 0.05, (iv) SNPs were retained only if they could be genotyped in at least 70% of the samples, and (v) SNPs were also pruned for population structure analysis according to linkage disequilibrium using the software Plink^26^.

#### Detection of putatively adaptive loci

Putatively adaptive loci were estimated independently from the linkage disequilibrium pruned SNP dataset using three genome scan programs (fsthet, pcadapt and OutFLANK), similar to Vaux et al.’s^19^. The expected heterozygosity (He) and divergence coefficient (Fst) were calculated using fsthet based on Plink to filter the adaptive loci based on the eastern and western groupings. fsthet is based on the percentile distribution of the He segments Fst, and we extracted the 1% quartile of each segment as the bounds of candidate adaptive loci. The loci corresponding to the 1% quartile of Fst which is larger than the 1% quartile are the candidate adaptive loci. Default settings were used for OutFLANK with q value < 0.05 based on the same groups, and only one loci was selected. So we used a screened top 0.5% of the loci with the smallest p-value as candidate adaptation loci. In pcadapt, we applied the default settings, including an alpha value of 0.1, and only the first two PCs were analyzed without any prior population groupings. The qvalue 2.12.0 R package was used to estimate FDR for pcadapt.

#### Population genetic differentiation

After filtering loci, genetic variation was investigated for each population at putatively adaptive and neutral loci. Observed (Ho) and expected (He) heterozygosity, inbreeding coefficient (Fis)—hereafter described as the within subpopulation Hardy–Weinberg deviation index—were calculated, which may reflect inbreeding, Wahlund effects, or other processes affecting heterozygosity. Deviations from Fis = 0 were tested using chi-square analysis, with H_O_ and H_E_ converted to frequency counts by multiplying by subpopulation sample sizes and summing across groups, implemented in the “chisq.test” function in R^13^. Pairwise Fst were measured by software Stacks 2.66^27^. We performed 10,000 permutations for significance at putatively adaptive loci in Arlequin 3.5.2.1^28^.

The Neighbor-Joining (NJ) phylogenetic tree was constructed using the software FastTree^29^. Based on two datasets, a neighbor-joining tree was generated by FastTree with -gtr -gamma model and 1000 bootstraps.

The unsupervised maximum-likelihood clustering algorithm implemented in ADMIXTURE^30^ was used to cluster each ancient genome in the investigated population. Initial clustering was performed for k = 1 to k = 20 ancestral clusters with default settings, we determined K by minimizing cross validation error (CV) as per Alexander and Lange^30^ and Anderson et al.^13^. To maximize the accuracy of the initial clustering, pruned SNPs (i.e., subset of adaptive SNPs) were used for structure analysis. To visualize the genetic relationships among samples, we performed principal component analysis (PCA) based on pruned SNPs using Plink^26^. Analyses were also carried on neutral SNPs. NewHybrids^31^ was used to identify hybrid individuals between two populations based on putatively adaptive loci with default settings.

#### Functional annotation and enrichment analysis

To identify the functional implications of adaptive loci, variant annotation was performed using SnpEff^25^, which provided transcript-level information for genes associated with candidate loci. Protein sequences encoded by these transcripts were aligned to pre-downloaded reference databases using diamond^32^ and HMMER^33^, facilitating the assignment of GO and KEGG annotation terms. A custom orgDB annotation database was then constructed using the AnnotationForge R package^34^ based on transcript-to-GO/KEGG mappings. Finally, GO and KEGG enrichment analyses were conducted using the clusterProfiler package^35^ and the custom orgDB database.

### Growth analysis

#### Vertebrae processing

The 32nd, or anterior and posterior vertebrae were chosen to estimate the age of fish. To obtain these vertebrae, the thawed vertebrae were boiled in water for approximately 10 min^36,37^. Muscle tissue was removed with a brush and knife, after which the vertebrae were separated along the gap between two vertebrae and the jelly was removed. The vertebrae were washed with water and used directly for the next staining process.

To achieve more distinct annual growth bands, we modified the alizarin red staining method of Zhou et al.^37^ by reducing the alizarin red concentration to 0.05%. The staining process varied with temperature and took at least 6 h in summer. After staining, vertebrae were dried and prepared for the observation of annual bands.

Vertebrae have been validated through comparisons of different hard parts in southern bluefin tuna^38^, and can be used to age more than ten years^39,40^. This method can accurately estimate ages for young year groups; however, it may not affect the estimation of growth parameters in the absence of older age groups^41^. Age was estimated by counting the bands observed on the inner surface of vertebrae, and annual increments were considered as one consecutive groove and ridge^36^. Grooves form during periods of rapid growth in summer, while ridges develop during slower growth phases in winter^19^. Both anterior and posterior surfaces were used to interpret the total bands. For each vertebra, bands were interpreted by a principal reader (R1) and a less experienced reader (R2) with no biological information^37,40^. If there were no agreements, the third interpretation was conducted to determine the final result by R1 with access to the first two counts. An interval of 2 weeks was required before the third interpretation. Average percent error (APE) was used to estimate aging error among readers, while an APE of less than 10% was considered to indicate accuracy and precision^42^.

#### Length-weight relationship

The length-weight relationships were estimated using the formula:

1 $$\:\begin{array}{c}W=\:a\times\:{FL}^{b}\end{array}$$

where a and b are parameters of the power function determined by the method of least squares. Range of FL-W data was different for two regions; therefore, the FL-W relationship was compared both within the same class (75–105 cm) and across all samples from different regions using the emtrends() in R package emmeans^43^.

#### Growth models

Age was estimated from 228 western individuals ranging from 4 to 8 years, and 419 eastern samples ranged from 2 to 9 years. The APE among age readers was 7.90% from a total of 647 specimens, ranging from 64.6 to 104.9 cm. The Von Bertalanffy growth model^44^, the Logistic growth model^45^, the Gompertz growth model^46^ and the Richards growth model^47^ were fitted to the length-at-age data of albacore tuna using nonlinear least-squares regression. The form of these models was:

2 $$\:\begin{array}{c}{L}_{t}={L}_{\infty\:}\left(1-{e}^{-K\left(t-{t}_{0}\right)}\right)\end{array}$$

3 $$\:\begin{array}{c}{L}_{t}={L}_{\infty\:}{\left(1+{e}^{-K\left(t-{t}_{0}\right)}\right)}^{-1}\end{array}$$

4 $$\:\begin{array}{c}{L}_{t}={L}_{\infty\:}{e}^{-\left(\frac{1}{K}\right){e}^{-K\left(t-{t}_{0}\right)}}\end{array}$$

5 $$\:\begin{array}{c}{L}_{t}=\frac{{L}_{\infty\:}}{{\left(1+b\ast\:{e}^{-K(t-ti)}\right)}^{\frac{1}{b}}}\end{array}$$

where L_t_ is the fork length at age t in cm, L_∞_ is the asymptotic fork length in cm, the value of K is the relative instantaneous growth rate in year^− 1^, t is the age, and t_0_ is the theoretical age in the year when FL is zero; b is a dimensionless parameter; ti is age at the inflection point.

FLs of albacore, including those converted through the linear function, and age were used to fit models. Growth model parameters were computed using the R version 4.3.1 with the package FSA^43^. Four candidate models were evaluated for relative support by lowest AICc. The Akaike weight, *w*, of each model was calculated. Likelihood Ratio Test (LRT) was used to examine the area-specific growth functions for both all samples and samples from same age class (4-8years)^48^.

## Results

### Genetic population structure

Sequencing of 54 fish from two regions identified 7,754,088 SNPs after the quality filtering process. These SNPs were used to identify putatively adaptive loci by three genome scan programs. A total of 307 loci identified by all programs were selected for further analysis. For each region, Ho is lower than He, and the genetic diversity of the western population was higher than that of the eastern population (Table 1). The Fis values of two populations were 0.087 and 0.133. Deviation from zero was evaluated via a chi square test (Eastern: χ² = 0.086, df = 1, *p* = 0.769; Western: χ² = 0.024, df = 1, *p* = 0.876), indicating no significant departure from Hardy–Weinberg expectations. For the neutral dataset, both populations exhibited similar levels of genetic diversity, with no significant deviation(Eastern: χ² = 0.001, df = 1, *p* = 0.972; Western: χ² = 0.002, df = 1, *p* = 0.968).

**Table 1:** Genetic diversity for each population

SNPs	Region	*N*	Ho	He	Fis
Adaptive	Western	23	0.409	0.440	0.087
	Eastern	31	0.296	0.339	0.133
Neutral	Western	23	0.282	0.275	0.006
	Eastern	31	0.281	0.276	0.006

Observed (Ho) and expected (He) heterozygosity and Fis were calculated based on putatively adaptive and neutral loci

**Fig. 2:**
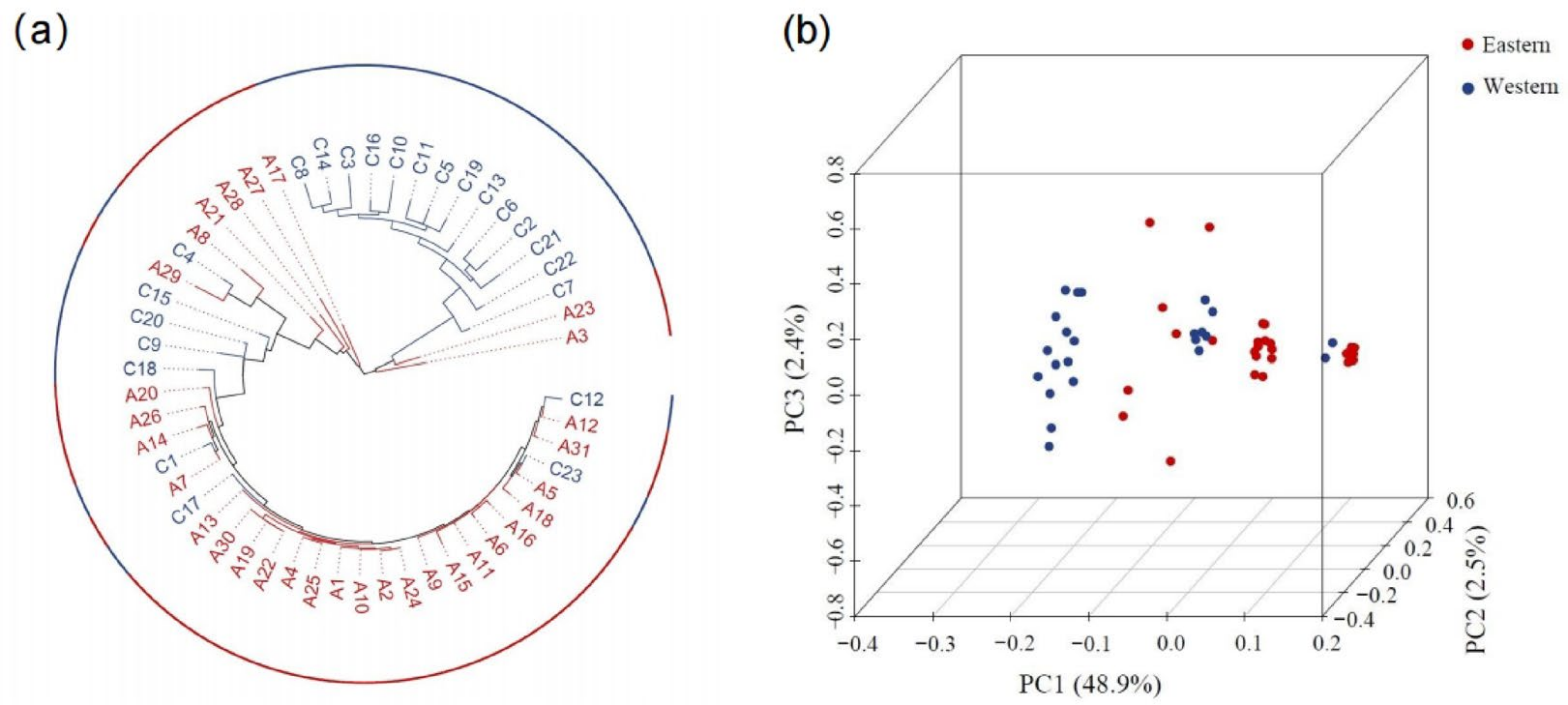
Genetic clusters. (**a**) Neighbor-Joining phylogenetic tree and (**b**) PCA cluster analysis were used to detect clusters based on selected SNP loci. Eastern and western samples were colored in red and blue, respectively.

Using 307 putatively adaptive loci, preliminary population structure between two regions was observed. The pairwise Fst between two populations was 0.138 (*P* < 0.0001). The phylogenetic tree detects two genetic branches (Fig. 2a), with one branch comprising two-thirds of the western individuals and the other consisting primarily of the eastern samples (Fig. 2a). Principal component axis 1 (Fig. 2b) clusters and separates more than half of the Western Pacific samples from the rest, while the remaining Western Pacific individuals intermix with those collected in the Eastern Pacific. Cross validation in ADMIXTURE analysis revealed two main genetic backgrounds (Supplementary Figs. S1 and S2), which may correspond to the differentiated eastern and western populations. However, reproductive isolation appears incomplete, as individuals with approximately 50% mixed ancestry (putative F₁ hybrids) and ~ 25% ancestry (likely backcrosses) were observed in both regions. These findings may indicate either limited interbreeding between two discrete spawning populations or partial gene flow within a shared spawning area constrained by reproductive barriers. According to NewHybrids, seven out of 23 individuals from the western population were classified as F1 hybrids with posterior probabilities exceeding 75%, and these individuals also exhibited intermediate ancestry proportions (0.4–0.6) in Admixture and clustered in the central region along PC1 (Fig. 2b; Table S3). Additionally, one individual was identified as a first-generation(F1) hybrid with a posterior probability of 67%. The remaining individuals were more likely assigned to second-generation hybrids (F2), based on their posterior probabilities.

Panmixia at putatively neutral SNP was detected for all analyses. In the PCA, two discrete clusters are not evident; however, a slight tendency is observed, with western individuals more often occupying negative values along PC1 (2.0% of the variance), while eastern individuals tend toward positive values (Supplementary Fig. S5). NJ phylogenetic tree revealed no clear clusters among all samples (Supplementary Fig. S5). ADMIXTURE software identified K = 1, indicating one single genetic population in the South Pacific (Supplementary Figs. S6 and S7), and most samples exhibited nearly equal admixture proportions when K = 2 and 3 was tested.

All putatively adaptive SNPs were successfully aligned to genes, resulting in 554 functional annotations. Of these, 548 annotations matched fish DNA sequences in GenBank, including 503 aligned to South Bluefin Tuna sequences. A total of 309 annotations were enriched in GO terms, while 170 matched KEGG pathways. The GO enrichment results show significant enrichment in muscle structure and contraction-related functions, mitochondrial processes, DNA repair, and neural signaling pathways (Fig. S3). The KEGG enrichment analysis shows significant enrichment in pathways related to protein export, nicotine addiction, fatty acid metabolism, steroidogenesis, synaptic signaling, and DNA repair (Fig. S4).

### Growth analysis

#### Length-weight relationship

**Fig. 3:**
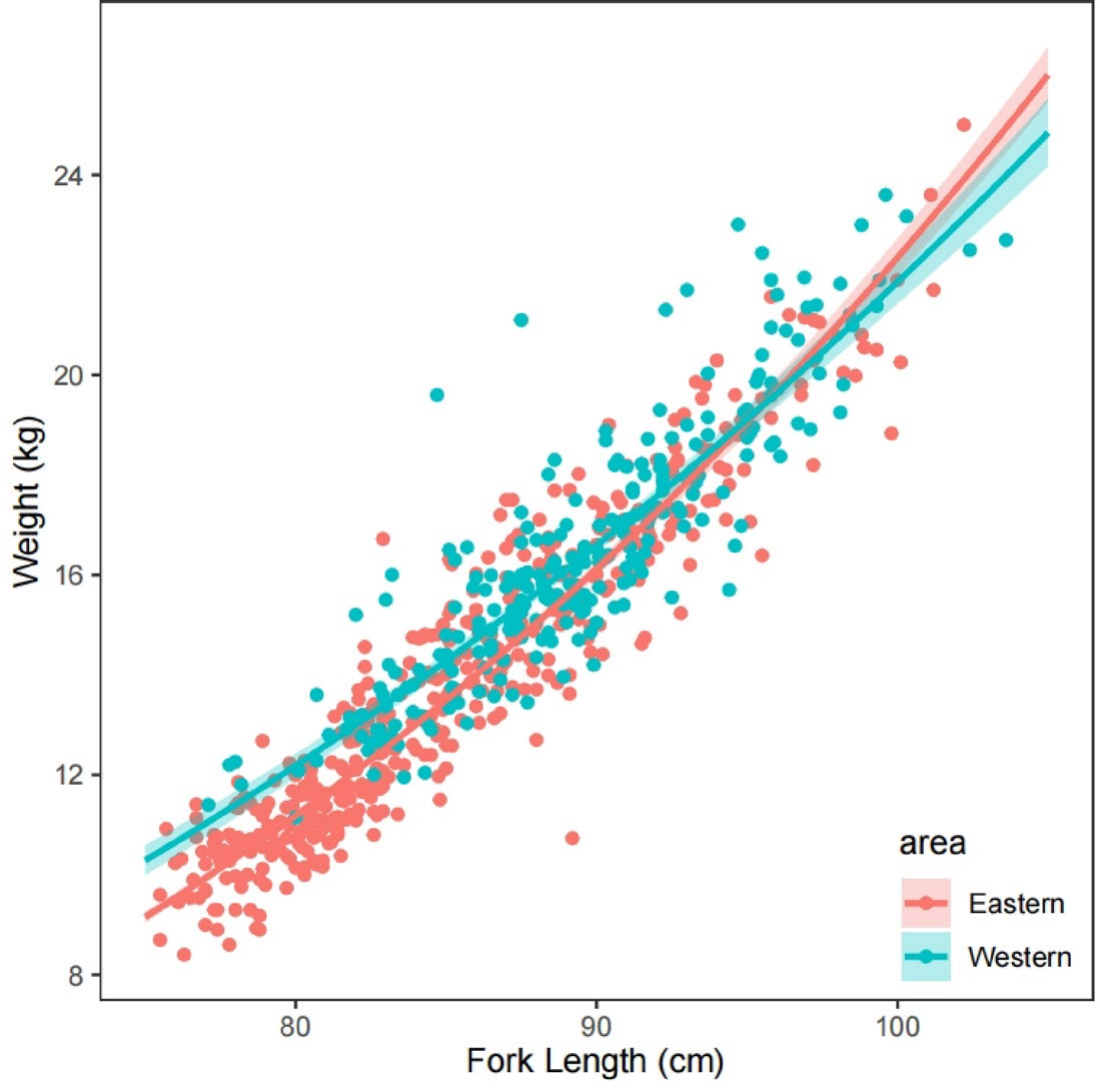
Length-weight relationships of albacore in two regions with same length range (75–105 cm FL). Eastern and western samples were colored in red and blue, respectively.

The length-weight relationships are significantly different between geographic regions in slopes and intercepts (Table 2). The b value indicates negative and positive allometric growth of western and eastern populations, respectively, and eastern individuals can grow larger at the same length (Fig. 3). Length-weight relationships of western samples show that there is no significant difference in 2023 and 2024 (*P* < 0.0001, Table 3), but comparison of eastern samples shows a difference between intercepts. Individuals caught in 2021 and 2022 were not used to compare the year difference due to the limited data. Regional variations may be not related to catch pressure, which appears to be of similar magnitude across regions (Supplement Fig. S8).

**Table 2:** Fork Length-weight relationships of Albacore for different year groups with different FL ranges

Area	year	*n*	FL range	a	b	*R* ^2^
Eastern	2021–2024	457	60–105	1.4*10^-5 (3*10^-6)	3.10 (0.05)	0.91
	2023	86	65–105	1.9*10^-5 (8*10^-6))	3.04 (0.09)	0.93
	2024	311	70–100	3.3*10^-5 (1.1*10^-5)	2.91 (0.07)	0.84
Western	2022–2024	457	75–105	1.26*10^-4 (4.6*10^-5)	2.62(0.081)	0.80
	2023	128	75–105	1.96*10^-4 (9.8*10^-5)	2.52 (0.11)	0.77
	2024	100	75–105	4.7*10^-5 (2.4*10^-5)	2.84 (0.11)	0.87
All samples	2021–2024	715	60–105	1.5*10^5 (3*10^-6)	3.09 (0.04)	0.90

N is the number of individuals, a is the constant, b is the allometric coefficient, and R^2^ is the coefficient of determination. The numbers in brackets represent the standard error of the parameter

**Table 3:** Comparisons of parameters of length-weight relationship

Groups	Parameters	coef	DF	t	*p*
2023 Western-2024 Western	slops	-0.32 (0.17)	225	-1.89	0.0601
	intercepts	-0.00 (0.00)	226	-0.175	0.8614
2023 Eastern-2024 Eastern	slops	0.13 (0.11)	393	1.14	0.26
	intercepts	0.01 (0.00)	394	2.091	0.04
Western-Eastern (75–105 cm)	slops	0.44 (0.10)	689	4.35	< 0.0001
	intercepts	-0.02 (0.00)	690	-6.325	< 0.0001
Western-Eastern	slops	0.48 (0.10)	711	4.964	< 0.0001
	intercepts	-0.02 (0.00)	712	-6.109	< 0.0001

Parameters of 2023 and 2024 data collected from the same region were compared, as well as parameters estimated from all samples and for the same range (75–105 cm FL) between regions. Coef represents estimated effect size or group difference; DF is degrees of freedom; t is the test statistic; and p indicates significance, with *p* < 0.05 considered significant. Parameter comparisons with significant differences between groups are in bold, and the numbers in brackets represent the standard error of the parameter

### Growth pattern

Gompertz model was the best supported among all candidate models (w = 0.31), von Bertalanffy model and Logistic model provided substantial approximating description of the data (ΔAICc = 0.12, w = 0.29; ΔAICc = 0.06, w = 0.31; Table 4). The Richard model received less support with ΔAICc = 2.03 and Akaike weight of 0.11. The comparisons of growth between two regions with different models differed significantly (*P* < 0.001). Parameters of eastern samples showed a relatively faster growth rate (k = 0.29–0.42) and smaller theoretical maximum fork length (L_∞_=97.24-100.01; Supplementary Table S2). Therefore, samples from two regions cannot be combined to fit the growth parameters. For growth-parameter comparisons, we selected a subset of eastern samples matching the age distribution of western samples. There were significant differences between parameters of two datasets. Richard model was not compared due to the convergence of western samples.

**Table 4:** Parameters estimated from four models for all samples included converted FL

Model	L_∞_	k	t_0_	b	ti	AICc	ΔAICc	w
VB	100.28 (2.37)	0.32 (0.05)	-1.05 (0.51)			3967.58	0.09	0.29
Logistic	97.91 (1.71)	0.45 (0.06)	0.61 (0.23)			3967.68	0.11	0.29
Gompertz	98.92 (1.98)	0.39 (0.05)	-0.08 (0.33)			3967.58	0	0.31
Richard	99.00 (4.73)	0.38 (0.25)		-0.07 (3.96)	-0.14 (3.23)	3969.61	2.03	0.11

Parameters L_∞_, k, t_0_, b and Ti are estimated from growth models. AICc is the small-sample bias-corrected form of akaike’s information criterion, ΔAICc is the Akaike difference, and w is the Akaike weight. The numbers in brackets represent the standard error of the parameter

## Discussion

Although highly migratory marine species often experience extensive gene flow in the absence of geographic barriers, population structure can still emerge^49^. Environmental gradients create ecological boundaries that restrict dispersal and drive adaptive evolution, shaping subpopulation dynamics. Given the species’ wide geographic distribution and habitat heterogeneity, habitat specific analyses are essential to accurately characterize population structure and avoid biased management^18^. This study utilizes SNPs derived from genome-wide resequencing and growth data from a comprehensive sample spanning both the eastern and western South Pacific to identify the population structure of albacore. Our results reveal that individuals in the western region can grow to larger sizes, with potential differentiation occurring in the South Pacific likely driven by environmental adaption.

Our study identified preliminary genetic differentiation in South Pacific albacore using adaptive SNPs selected based on eastern and western groupings (Fst = 0.138, *p* < 0.00001). While these loci were chosen to maximize inter-group contrast and may slightly overestimate genome-wide divergence, combining multiple analytical approaches revealed two main genetic branches corresponding to the WCPO and the eastern Pacific, despite high connectivity between regions.

These findings align with Anderson et al.^13^, who detected genetic differentiation using adaptive loci, whereas neutral markers revealed no structure, and complement previous reports showing weak or non-significant differentiation with neutral loci^15,18^. This highlights that adaptive SNPs provide critical resolution for detecting biologically meaningful differentiation in highly migratory species such as *Thunnus alalunga*.

Adaptive variation is often driven by natural selection and closely linked to environmental changes. Significant environmental differences among water bodies can act as geographic barriers, promoting genetic differentiation. For example, the south and north Pacific albacore are separated by the equator, yet some connectivity persists between the two stocks^19^, whereas relatively consistent environmental conditions in the WCPO may facilitate gene flow^13^. Environmental variation can either enhance or restrict connectivity, ultimately influencing allele frequencies^50^. Similar effects are observed in benthic species, where local populations exhibit variation in growth rates, mortality, and genetic variation^51^.

In the western and eastern South Pacific, oceanographic features may form partial barriers to migration^52^; however, these are less pronounced than at the equator, allowing some individuals to migrate and maintain connectivity^53^. Our analysis reveals weak population structure and admixture in certain individuals, suggesting that connectivity between the two regions may persist over the long term. Notably, a few eastern individuals clustered with western individuals but still showed genetic differences (Fig. 2a), indicating the possible presence of a separate genetic population within the South Pacific. The mechanisms behind this connectivity— whether adult migration, larval dispersal, or limited regional movement— remains unclear^54^. These observations further emphasize why adaptive loci are particularly effective in detecting subtle differentiation. While all SNPs in our study were annotated, the influence of natural selection or fishing pressure on these patterns requires further investigation. Overall, our results demonstrate that adaptive variation is essential for uncovering biologically meaningful population structure, providing critical insights into evolutionary dynamics, demographic patterns, and conservation management in highly migratory marine species.

The relationship between site fidelity and migration after maturity remains unclear for the South Pacific albacore. Juveniles are distributed at high latitudes, later dispersing to low latitudes^13,54^. Tagging data supported the larval dispersal from high latitudes but provided limited information on longitudinal migration patterns of adults^54^. The known spawning ground in the WCPO, located between 10–25°S, suggests that albacore aggregate for reproduction upon reaching sexual maturity, regardless of prior dispersal patterns. However, the limited overlap between our sampling times and spawning period likely minimized the possibility of mixture^17^. Several larval albacore were collected from tows in the eastern region^55^, and predictions of larval distribution also suggest potential occurrences in the eastern subtropical regions^56^. The eastern Pacific barrier hinders connectivity of coral larval and tropical shallow-water species^57,58^. The larval stage of albacore may be unable to cross this barrier, potentially contributing to variation in the spawning grounds inferred from trace elements^16^ or to the genetic differentiation in our study. According to different weights of mature gonads in different region^17^, there may be geographic separation of spawning groups. Reproduction may be driven by site fidelity^59,60^. For example, Atlantic bluefin tuna populations return to their natal spawning grounds despite feeding in overlapping areas. Separate spawning regions reduce the gene flow and increase population-specific genetic variation. Different environmental factors can result in variations in growth rates and life history^61^. Spawning typically occurs during the austral spring and summer with peak months from October to December^17^. Temporal variation can also be observed within a single population; for instance, Atlantic bluefin tuna (*Thunnus thynnus*) in the eastern Mediterranean Sea spawn earlier than those in the western region^62^. Further analysis is required to determine whether differences in spawning periods are related to population differentiation.

The comparison of growth parameters may provide preliminary evidence for population structure. Williams et al.^9^ reported that albacore tend to grow larger at more easterly longitudes, but their study was limited to 130°W and did not assess growth variation in more eastern regions. Based on their observation of decreasing growth trajectories for males east of 170°W^9^, our results indicate that individuals in the eastern Pacific are smaller than those in western regions, potentially extending this trend to previously unstudied region. However, we only compared growth parameters and did not perform fine-scale growth estimation. As a highly migratory species, spatial structure likely explains only partial of the observed variation in growth. Therefore, region-specific growth assessments may provide more accurate and representative estimates, and careful attention should be given to the definition of regional boundaries.

Spatial variation in albacore growth may also reflect environmental factors, fishing pressure, or genetic differentiation^9^. In our study, samples were collected from longline vessels, so gear selectivity is unlikely to bias length distribution between regions. Observed differences in size may partially result from variation in life-history stages: the age range of western samples aligns with sexual maturity^63^, as all individuals were older than four years, while similar, immature albacore are more likely to be caught at high latitudes^9^. Thus, some of the observed size differences may reflect age composition rather than true growth differences.

Variation in the length-weight relationship can also be influenced by age, since different life stages exhibit distinct growth patterns^64^, especially following dietary shifts^65^. To reduce potential bias, we restricted our analyses to individuals with fork lengths of 75–105 cm, representing mostly sexually mature fish. The similar spatial distribution of longline catches across regions suggests that fishing pressure had limited impacts on the observed growth differences. Overall, while some variation may reflect differences in age composition, genuine spatial differences in growth cannot be entirely ruled out.

Environmental change can induce variations in fish growth patterns. Sea surface temperature (SST) has the most significant effect on fish growth and can directly affect individual growth rate^66^. By analyzing vertebrae from the historical era, incremental changes were found to reflect the positive effect of climate warming on the growth of Atlantic bluefin tuna^67^. This result is similar to the growth rate trend in southern bluefin tuna during the latter half of that century^68^. Environmental changes may directly influence the growth rate of fish and potentially cause mutations at certain genomic loci, thereby enhancing the genotype’s adaptability to environmental conditions. Both environmental and genotypic changes simultaneously influence the growth process of fish, making it difficult to separately assess their impacts, especially for highly migratory species. Although genomic analysis suggests the presence of two genetic clusters, it is not feasible to estimate growth separately for each cluster. Doing so would require extensive additional sampling and analyses, and the clusters do not correspond fully to distinct geographic regions, making such assignments unreliable. Some studies attempt to link genetic loci to environmental factors to infer population structure, but this approach is not well-suited for highly migratory fish. Consequently, growth estimates for the two genetic clusters can only be meaningfully compared across geographical regions, using greater spatial separation to reduce the influence of gene flow between groups.

Different subpopulations may exhibit distinct population dynamics^1^. Incorrect population structure in stock assessment can lead to inaccurate estimates of local populations, potentially undermining the effectiveness of management measures. Current stock assessment processes incorporate details like fishery dynamics, spatial structure and migration, but often overlook heterogenous dynamics and demographics influenced by population structure and temporal and spatial biological variation by climate change^14^. A lack of understanding of population structure in stock assessment may produce biased total allowance catch and harvest control rules, potentially leading to overfishing. Subpopulation collapse can easily occur in mixed-stock fisheries^2^, particularly when it goes unrecognized. Population depletion caused by this mismatch is difficult to rebuild, which will cause changes in biological characteristics and a reduction in genetic diversity. Additionally, management units often represent only a portion of a self-sustainable population, making it challenging to fully exploit and implement effective conservation measures without a comprehensive understanding^69^. This study integrates intrinsic and phenotypic factors to provide critical evidence for the existence of subpopulations and to enhance the accuracy of stock assessments. Our results reveal a mixed stock comprising potentially adaptive differentiated populations, suggesting a mixture of adult albacores under selection of environmental variations or fishing. Therefore, the definition of management units should consider both population structure and ecological drives.

Boundaries and biological characteristics of South Pacific albacore subpopulations remain poorly defined, and the evidence for differentiation is limited and sometimes inconsistent. Reliance on demographics and dynamics estimates derived mainly from WCPO samples risks biasing abundance and productivity assessments for other regions. In contrast, our genetic and growth results point to population structure that may not be captured by WCPO-centric assessments. Furthermore, Simulation of Spatial Ecosystem And Population Dynamics Model (SEAPODYM) projections indicated decreased spawning levels for southern albacore in the current ground and a southward shift in habitat^4,70^, underscoring the need to broaden the spatial scope of data collection. Collecting biologically and environmental data across a wider geographic range is therefore essential to resolve these uncertainties and to integrate regionally relevant parameters into stock assessments and conservation planning. Temporal factors such as spawning timing and post-spawning aggregation should also be considered, since populations may concentrate in distinct areas after reproduction; for example, Atlantic bluefin tuna have separate western and eastern spawning grounds while sharing feeding grounds^71^.

## Conclusion

Our study provides convergent evidence from independent genetic and growth analyses for subtle population structure in South Pacific albacore. Using adaptive SNPs, we detect genetic differentiation between western (WCPO) and eastern Pacific samples, while growth analyses reveal significant regional differences: western individuals tend to reach larger sizes but exhibit slower growth rates. However, we cannot yet disentangle whether these growth differences are driven primarily by genetic divergence or by environmental and life-history factors.

To resolve these uncertainties, future work should combine broader spatial sampling with integrative approaches —including targed age-structured growth estimation, genome scans, otolith chemistry, and tracking studies— to identify the drivers of differentiation and clarify connectivity. Finally, population dynamics and management scenarios should be explicitly tested (e.g. through simulation or spatially explicit stock assessments) to evaluate risks arising from mismatches between biological units and current management boundaries and to inform more robust conservation and fisheries strategies.

## Supplementary Information

Below is the link to the electronic supplementary material.

Supplementary Material 1

## Data Availability

The raw paired-end sequencing data for albacore generated in this study have been deposited in the Genome Sequence Archive (GSA) of the China National Center for Bioinformation (CNCB) under accession number CRA026790 (https://ngdc.cncb.ac.cn/gsa/browse/CRA026790).
