# Supplementary material for: Unveiling population structure in South Pacific albacore: insights from genetics and growth
Source: Sci Rep. 2025 Oct 17;15:36451. doi: 10.1038/s41598-025-20480-1 (PMC12534426; doi:10.1038/s41598-025-20480-1)
Supplement: Supplementary file 1 — Supplementary Material 1 [file 41598_2025_20480_MOESM1_ESM.docx]

**Unveiling Population Structure in South Pacific Albacore: Insights from Genetics and Growth**

Dongqi LU1, Qinqin LIN1, Feng WU1, Jiangfeng ZHU1,2,*, Fan ZHANG1,2,*

1. College of Marine Living Resource Sciences and Management, Shanghai Ocean University, Shanghai 201306, China;

2. Key Laboratory of Sustainable Exploitation of Oceanic Fisheries Resources, Ministry of Education, Shanghai 201306, China

Table S1 An overviews of the samples used for each analysis. The FL- W relationships were estimated using both measured fork length (FL) and total weight (W) data from 30, 128, 100 western samples collected in year 2021, 2023 and 2024, 56, 187 and 179 eastern samples from 2021 to 2023, respectively. For eastern samples subject to bleeding treatment, 13, 139, 308 samples with both FL and anal length (AL) measured from 2022 to 2024 were used to construct a linear conversion model to obtain FL data, primarily for estimating growth parameters. A total of 228 western and 419 eastern samples were successfully aged. Additionally, 23 western and 31 eastern samples harvested from May to August 2021 was sequenced and analyzed.

| Region | year | FL-AL Conversion | FL-W analysis | Growth function | Genetic analysis |
| --- | --- | --- | --- | --- | --- |
| Western | 2021 |  | 30 | 12 | 23 |
|  | 2023 |  | 128 | 148 |  |
|  | 2024 |  | 100 | 68 |  |
| Eastern | 2021 |  | 47 | 53 | 31 |
|  | 2022 | 13 | 13 | 187 |  |
|  | 2023 | 139 | 86 | 179 |  |
|  | 2024 | 308 | 307 | 0 |  |

Table S2 Comparison of parameters across four models. The parameters of eastern samples indicated a relatively faster growth rate (k = 0.27–0.39) and a smaller theoretical maximum fork length (L∞ = 98.36–101.7; Table S2). Significant differences were observed in the parameter estimates across the Von Bertalanffy, Logistic, and Gompertz growth models for both the entire age range and the same age range. The Richard model was excluded from comparison due to convergence issues with the western sample data.

| **Model** | **Region** | **L∞** | **k** | **t0** | **b** | **ti** | **D** | **p** |
| --- | --- | --- | --- | --- | --- | --- | --- | --- |
| Gompertz | Western | 124.19 | 0.12 | -4.03 |  |  | 55.62(269.69) | **<0.0001(<0.0001)** |
|  | Eastern | 99.42 | 0.36 | -0.32 |  |  |  |  |
| Logistic | Western | 118.71 | 0.16 | -1.67 |  |  | 55.75(269.62) | **<0.0001(<0.0001)** |
|  | Eastern | 97.24 | 0.42 | 0.46 |  |  |  |  |
| VB | Western | 133.41 | 0.08 | -8.73 |  |  | 55.57(269.95) | **<0.0001(<0.0001)** |
|  | Eastern | 100.01 | 0.29 | -1.41 |  |  |  |  |
| Richard | Western | 97.53 | 0.84 |  | 13.41 | 3.63 |  |  |
|  | Eastern | 97.79 | 0.39 |  | 0.50 | 0.10 |  |  |

Table S3 NewHybrids assignment. seven out of 23 individuals in western population were classified as F1 hybrids with posterior probabilities exceeding 75%,while one individual in eastern population was identified as a first-generation(F1) hybrid. The remaining individuals were more likely assigned to second-generation hybrids (F2). F1×Western and F1×Eastern represent backcrosses to the Western and Eastern populations, respectively.

| Sample | Western | Eastern | F1 | F2 | F1×Western | F1×Eastern |
| --- | --- | --- | --- | --- | --- | --- |
| A1 | 0 | 0.29656 | 0 | 0.70343 | 0 | 0.00001 |
| A2 | 0 | 0.29656 | 0 | 0.70343 | 0 | 0.00001 |
| A3 | 0 | 0 | 0 | 0.7275 | 0.2725 | 0 |
| A4 | 0 | 0.29656 | 0 | 0.70342 | 0 | 0.00002 |
| A5 | 0 | 0 | 0 | 0.70345 | 0 | 0.29654 |
| A6 | 0 | 0 | 0 | 0.70315 | 0.00031 | 0.29654 |
| A7 | 0 | 0 | 0.00001 | 0.7033 | 0 | 0.29669 |
| A8 | 0 | 0 | 0 | 0.99996 | 0.00004 | 0 |
| A9 | 0 | 0.29656 | 0 | 0.70342 | 0 | 0.00002 |
| A10 | 0 | 0.29656 | 0 | 0.70343 | 0 | 0.00001 |
| A11 | 0 | 0 | 0.00668 | 0.69674 | 0.00002 | 0.29656 |
| A12 | 0 | 0 | 0.04511 | 0.65833 | 0 | 0.29656 |
| A13 | 0 | 0.29656 | 0 | 0.70342 | 0 | 0.00002 |
| A14 | 0 | 0 | 0.00015 | 0.70329 | 0 | 0.29655 |
| A15 | 0 | 0 | 0 | 0.70343 | 0 | 0.29657 |
| A16 | 0 | 0 | 0.6714 | 0.03204 | 0 | 0.29656 |
| A17 | 0 | 0 | 0 | 0.99993 | 0.00007 | 0 |
| A18 | 0 | 0 | 0.00361 | 0.69985 | 0 | 0.29654 |
| A19 | 0 | 0.29654 | 0 | 0.70344 | 0 | 0.00002 |
| A20 | 0 | 0 | 0.00001 | 0.70316 | 0 | 0.29683 |
| A21 | 0 | 0 | 0 | 1 | 0 | 0 |
| A22 | 0 | 0.29654 | 0 | 0.70344 | 0 | 0.00002 |
| A23 | 0 | 0 | 0 | 0.72659 | 0.27341 | 0 |
| A24 | 0 | 0.29656 | 0 | 0.70342 | 0 | 0.00002 |
| A25 | 0 | 0.29656 | 0 | 0.70344 | 0 | 0 |
| A26 | 0 | 0 | 0.00002 | 0.70342 | 0 | 0.29656 |
| A27 | 0 | 0 | 0 | 0.99983 | 0.00017 | 0 |
| A28 | 0 | 0 | 0 | 0.73237 | 0.26763 | 0 |
| A29 | 0 | 0 | 0 | 0.86819 | 0.13181 | 0 |
| A30 | 0 | 0.29657 | 0 | 0.70333 | 0 | 0.0001 |
| A31 | 0 | 0 | 0 | 0.69464 | 0 | 0.30536 |
| C1 | 0 | 0 | 1 | 0 | 0 | 0 |
| C2 | 0.29643 | 0 | 0 | 0.7035 | 0.00007 | 0 |
| C3 | 0.29644 | 0 | 0 | 0.7035 | 0.00006 | 0 |
| C4 | 0 | 0 | 1 | 0 | 0 | 0 |
| C5 | 0.29646 | 0 | 0 | 0.70349 | 0.00005 | 0 |
| C6 | 0.29644 | 0 | 0 | 0.70348 | 0.00008 | 0 |
| C7 | 0.29642 | 0 | 0 | 0.70349 | 0.00009 | 0 |
| C8 | 0.29644 | 0 | 0 | 0.7035 | 0.00006 | 0 |
| C9 | 0 | 0 | 0.7496 | 0.2504 | 0 | 0 |
| C10 | 0.29645 | 0 | 0 | 0.70348 | 0.00007 | 0 |
| C11 | 0.29644 | 0 | 0 | 0.70348 | 0.00008 | 0 |
| C12 | 0 | 0 | 1 | 0 | 0 | 0 |
| C13 | 0.29646 | 0 | 0 | 0.70348 | 0.00006 | 0 |
| C14 | 0.29648 | 0 | 0 | 0.70348 | 0.00004 | 0 |
| C15 | 0 | 0 | 0.99999 | 0.00001 | 0 | 0 |
| C16 | 0.29646 | 0 | 0 | 0.70348 | 0.00006 | 0 |
| C17 | 0 | 0.29654 | 0 | 0.70344 | 0 | 0.00002 |
| C18 | 0 | 0 | 1 | 0 | 0 | 0 |
| C19 | 0.29646 | 0 | 0 | 0.70349 | 0.00005 | 0 |
| C20 | 0 | 0 | 1 | 0 | 0 | 0 |
| C21 | 0.29645 | 0 | 0 | 0.70348 | 0.00007 | 0 |
| C22 | 0.29647 | 0 | 0 | 0.7035 | 0.00003 | 0 |
| C23 | 0 | 0.29654 | 0 | 0.70344 | 0 | 0.00002 |


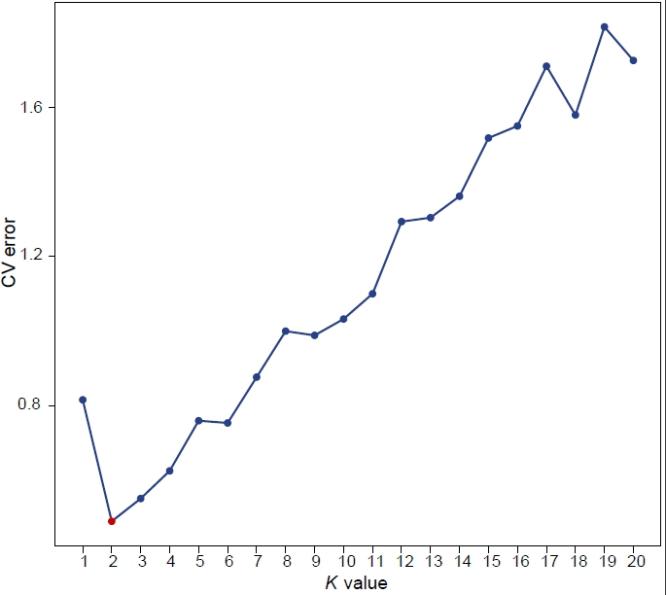


Figure S1 Population structure analyses cross validation errors for different K at putatively adaptive loci. Ancestral clustering analysis with k=1 to k=20 was performed using default settings and found that cross-validation error (CV) was lowest at k=2, and k=2 was identified as the optimal estimation


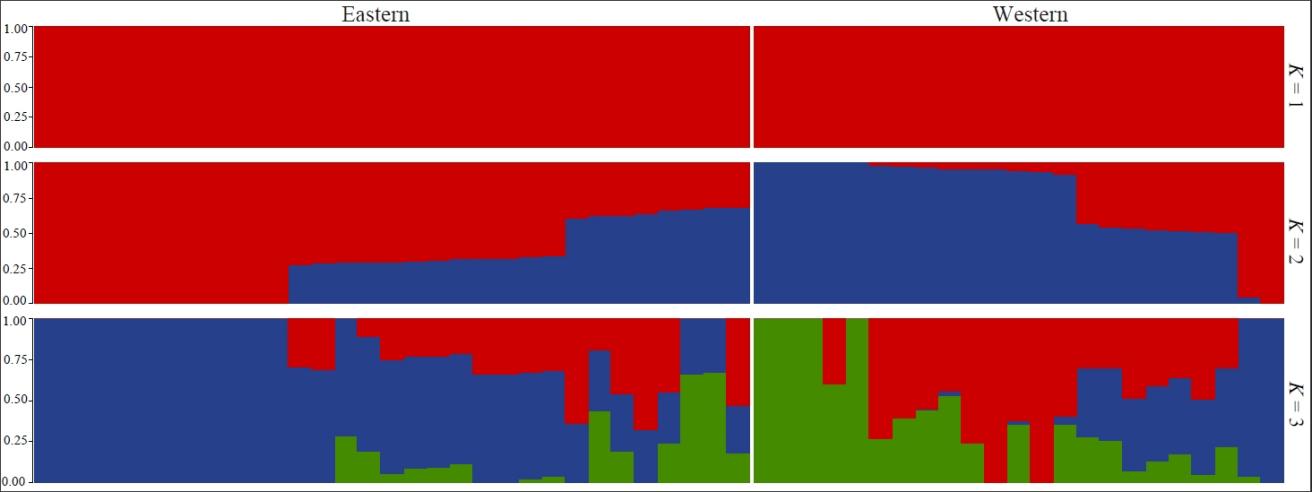
Figure S2 Admixture analysis for different K at putatively adaptive loci. It revealed genetic connectivity between western and eastern samples, which are consistent with findings from PCA analysis and the Neighbor-Joining phylogenetic tree. When k =3, the eastern population exhibits clearer differentiation from the western populations.


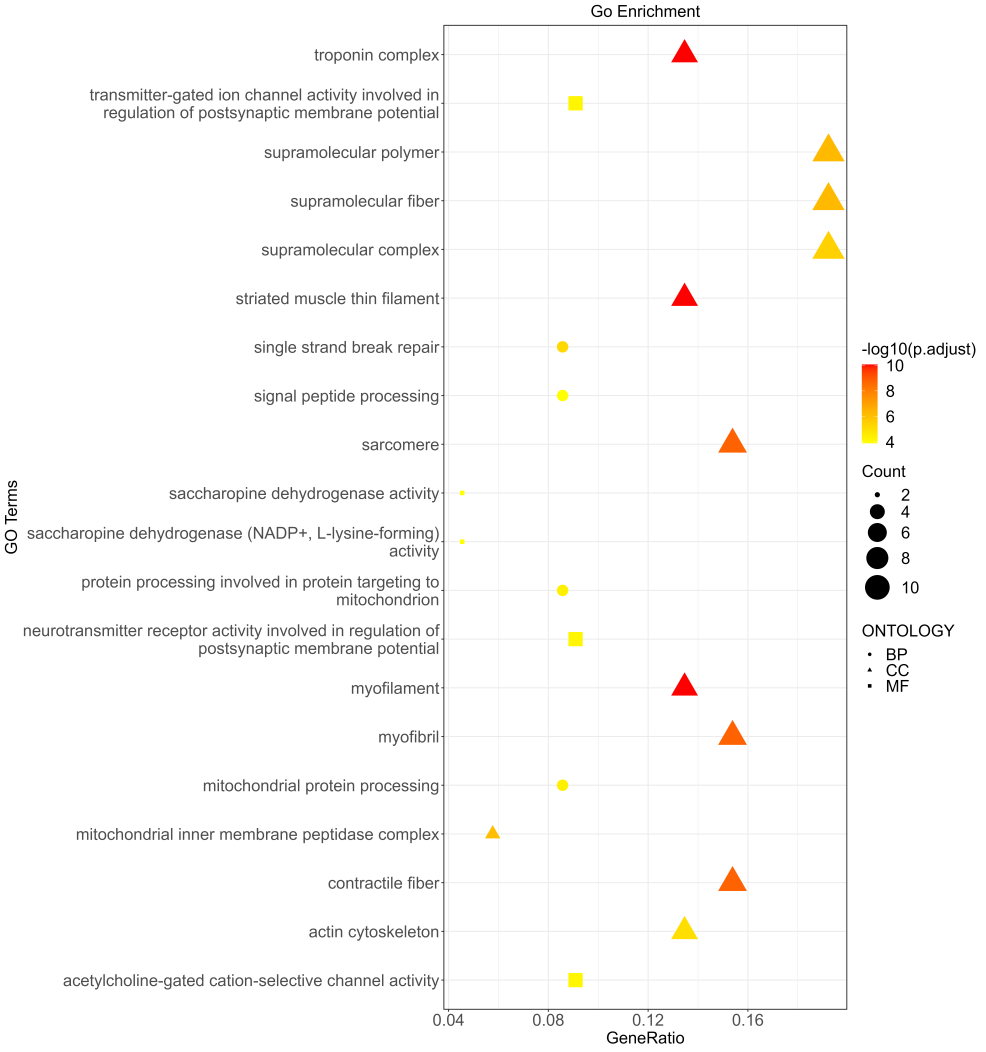


Figure S3 GO enrichment analysis of adaptive loci. Each point represents a significantly enriched GO term. The color intensity indicates the significance level (darker red corresponds to lower p-values). The size of the point reflects the number of genes associated with each GO term. Shapes denote the GO ontology categories: circles for Biological Process (BP), triangles for Cellular Component (CC), and squares for Molecular Function (MF).


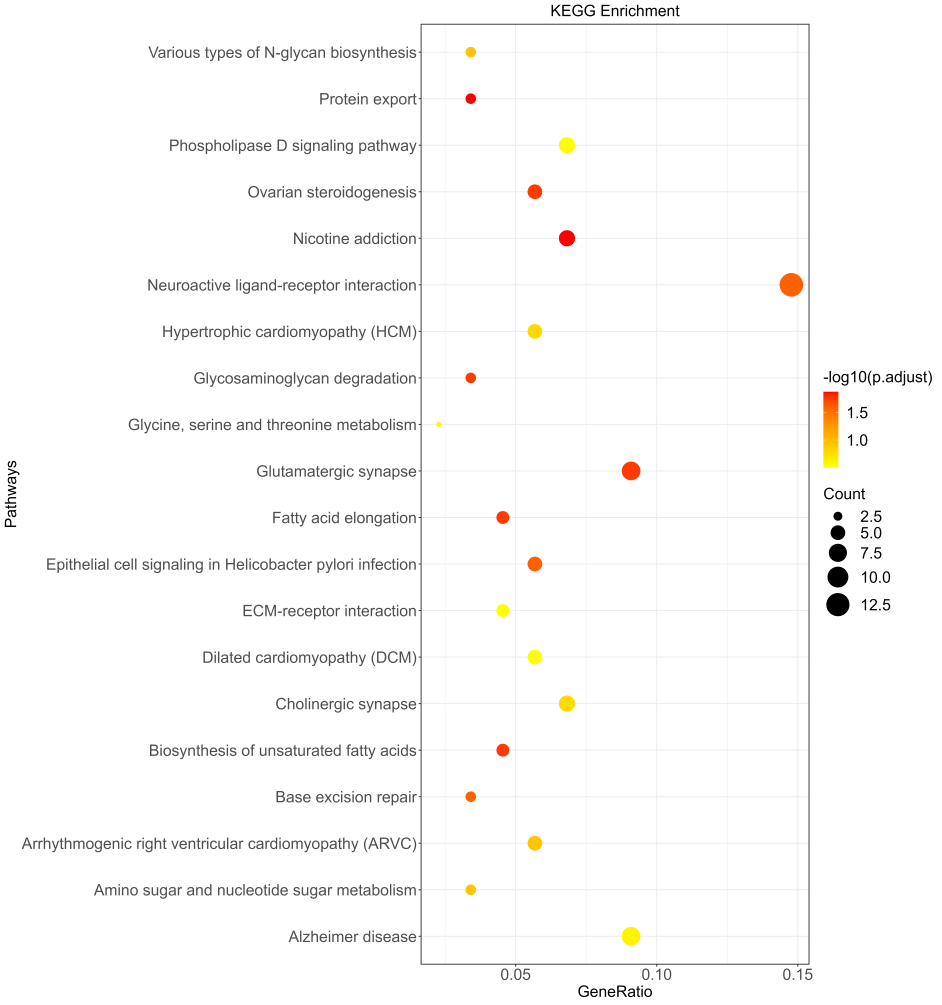


Figure S4 KEGG pathway enrichment analysis of adaptive loci[1-3]. Each point represents a significantly enriched KEGG pathway. The color intensity indicates the significance level (darker red corresponds to lower p-values). The size of the point reflects the number of genes associated with each pathway.


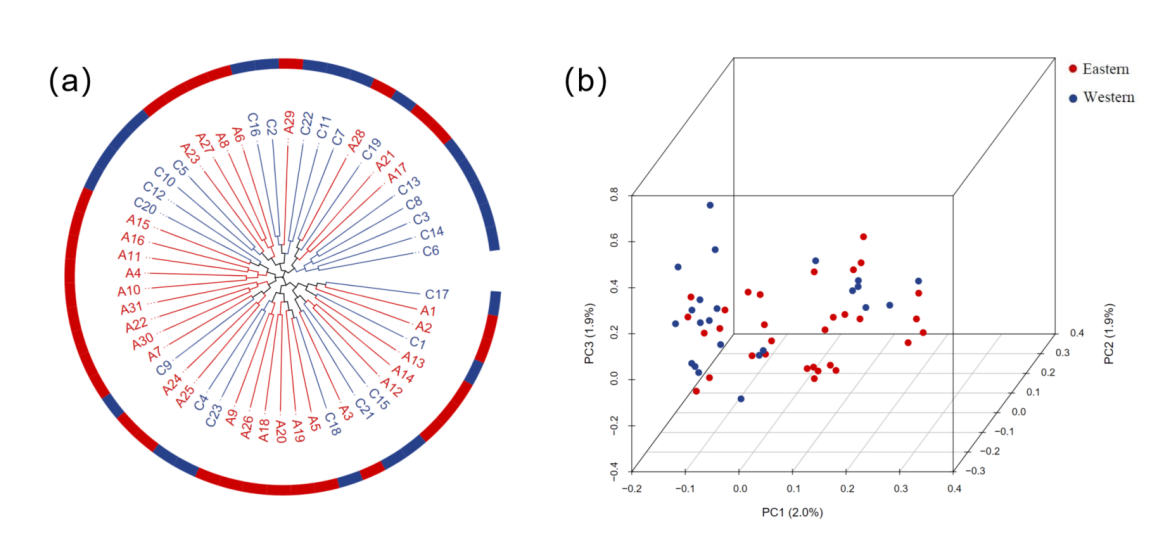
[1]

Figure S5 Genetic clusters of putatively neutral SNP. (a) Neighbor-Joining phylogenetic tree and (b) PCA cluster analysis were used to detect clusters without selected SNP loci. Eastern and western samples were colored in red and blue, respectively.


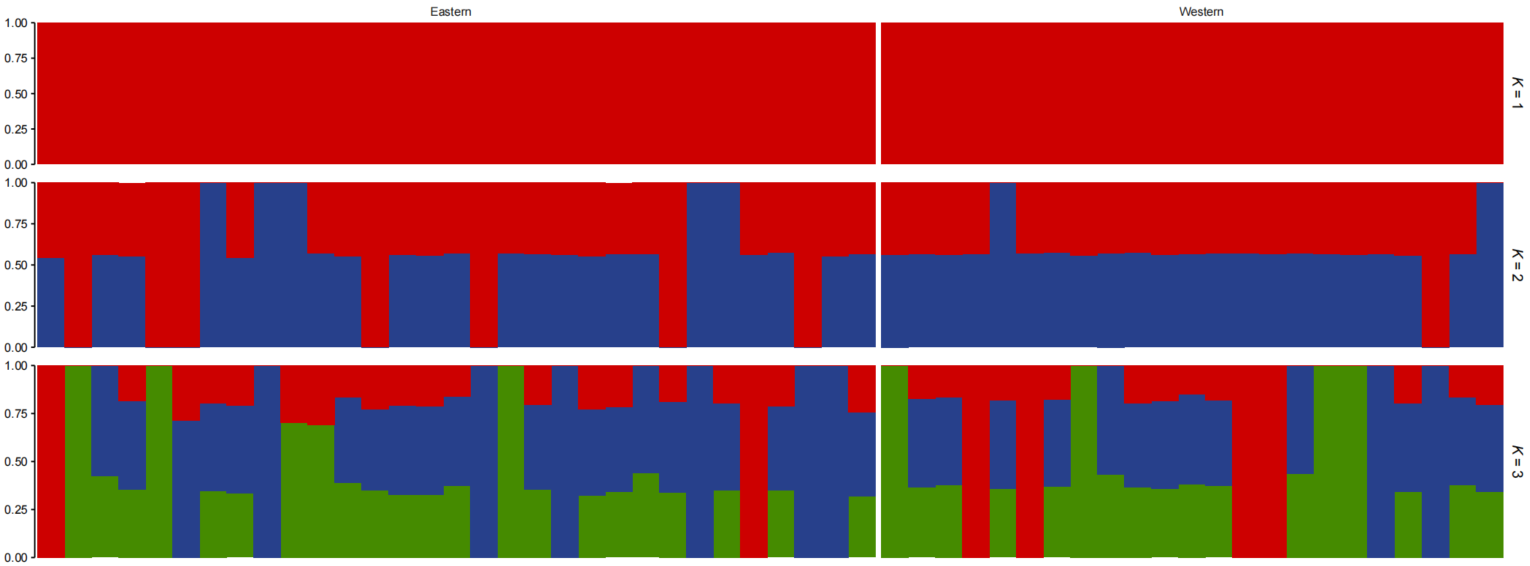


Figure S6 Admixture analysis for different K at putatively neutral loci. It revealed panamix between eastern and western populations.


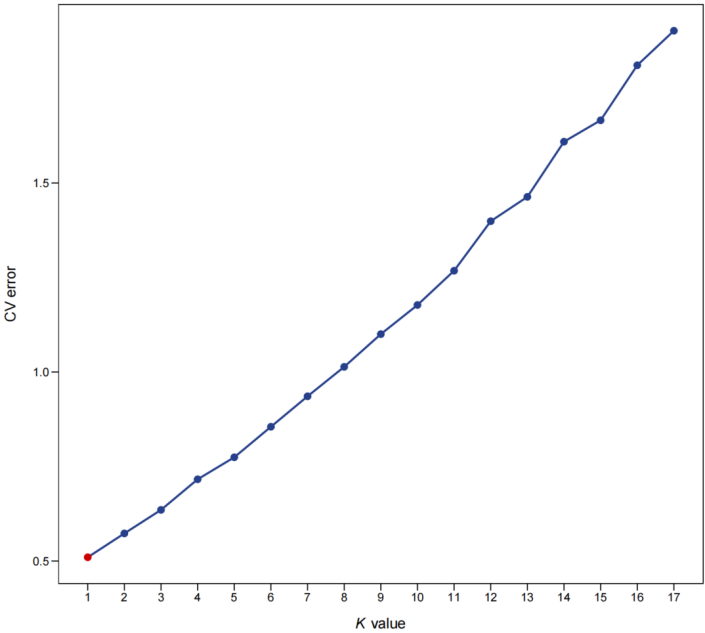


Figure S7 Population structure analyses cross validation errors for different K at putatively neutal SNP. Ancestral clustering analysis with k=1 to k=20 was performed using default settings and found that cross-validation error (CV) was lowest at k=1, which was identified as the optimal estimation


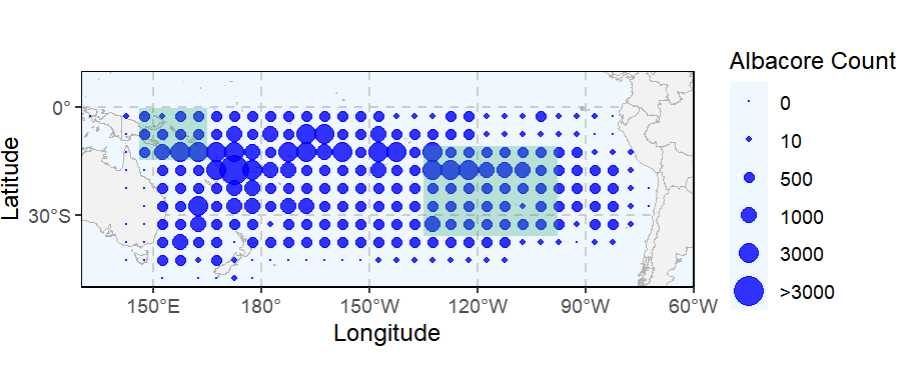


Figure S8 Spatial pattern of longline albacore catches (in thousand of individuals) from 2012-2023 in South Pacific. The green rectangles are the sampling regions in our study.

Reference

1. Kanehisa, M., Furumichi, M., Sato, Y., Matsuura, Y. & Ishiguro-Watanabe, M. KEGG: biological systems database as a model of the real world. Nucleic Acids Res. 53, D672-D677 (2025).
2. Kanehisa, M. Toward understanding the origin and evolution of cellular organisms. Protein Sci. 28, 1947-1951 (2019)
3. Kanehisa, M. & Goto, S. KEGG: Kyoto Encyclopedia of Genes and Genomes. Nucleic Acids Res. 28, 27-30 (2000).
